# Effect of human bone marrow mesenchymal stem cell-derived microvesicles on the apoptosis of the multiple myeloma cell line U266

**DOI:** 10.1007/s00432-024-05822-2

**Published:** 2024-06-08

**Authors:** Mona Vafaeizadeh, Saeid Abroun, Mina Soufi Zomorrod

**Affiliations:** Department of Hematology and Cell Therapy, Faculty of Medical Sciences, Tarbiat Modares University, Tehran, Iran

**Keywords:** Multiple myeloma, U266, Microvesicles, Mesenchymal stem cells, Cancer

## Abstract

**Background:**

Microvesicles are membraned particles produced by different types of cells recently investigated for anticancer purposes. The current study aimed to investigate the effects of human bone marrow mesenchymal stem cell-derived microvesicles (BMSC-MVs) on the multiple myeloma cell line U266. BMSC-MVs were isolated from BMSCs via ultracentrifugation and characterized using transmission electron microscopy (TEM) and dynamic light scattering (DLS). U266 cells were treated with 15, 30, 60, and 120 µg/mL BMSC-MVs for three and seven days and the effects of treatment in terms of viability, cytotoxicity, and DNA damage were investigated via the MTT assay, lactate dehydrogenase **(**LDH) assay, and 8‑hydroxy-2’-deoxyguanosine (8‑OHdG) measurement, respectively. Moreover, the apoptosis rate of the U266 cells treated with 60 µg/mL BMSC-MVs was also assessed seven days following treatment via flow cytometry. Ultimately, the expression level of *BCL2*, *BAX*, and *CCND1* by the U266 cells was examined seven days following treatment with 60 µg/mL BMSC-MVs using qRT-PCR.

**Results:**

BMSC-MVs had an average size of ~ 410 nm. According to the MTT and LDH assays, BMSC-MV treatment reduced the U266 cell viability and mediated cytotoxic effects against them, respectively. Moreover, elevated 8‑OHdG levels following BMSC-MV treatment demonstrated a dose-dependent increase of DNA damage in the treated cells. BMSC-MV-treated U266 cells also exhibited an increased apoptosis rate after seven days of treatment. The expression level of *BCL2* and *CCND1* decreased in the treated cells whereas the *BAX* expression demonstrated an incremental pattern.

**Conclusions:**

Our findings accentuate the therapeutic benefit of BMSC-MVs against the multiple myeloma cell line U266 and demonstrate how microvesicles could be of therapeutic advantage. Future in vivo studies could further corroborate these findings.

## Introduction

Multiple myeloma (MM) has been known as one of the most prevalent hematologic malignancies accounting for more than 10% of all diagnosed cases of blood-based cancers and around 1% of all diagnosed cancer cases (Rajkumar et al. 2014). Only in the United States, more than 30,000 individuals are annually diagnosed with MM, and around 10,000 lives are lost because of it (Siegel et al. 2021). The median age for individuals diagnosed with MM is 65 years old (with male patients having more cases than female patients) as it has been reported that MM is less prevalent in Caucasians in comparison with African Americans (Landgren and Weiss 2009; Kyle et al. 2003).

Over the past two decades, the treatment of MM has been significantly improved with the advent of novel treatment modalities. Conventional treatment for patients with MM include lenalidomide, bortezomib, and thalidomide alongside isatuximab (a CD38-specific monoclonal antibody), daratumumab (a CD38-specific monoclonal antibody), belantamab mafodotin (an antibody-drug conjugate specific for B-cell Maturation Antigen), pomalidomide (a TNF-α inhibitor), selinexor (an XPO1 inhibitor), carfilzomib (a protease inhibitor), elotuzumab (a humanized IgG1 SLAMF7-specific monoclonal antibody), ixazomib (a proteasome inhibitor), and idecabtagene vicleucel and ciltacabtagene autoleucel (both B-cell Maturation Antigen-redirected chimeric antigen receptor (CAR)-T products) (Yang et al. 2020; Cowan et al. 2022; Safarzadeh Kozani et al. 2022). Patients are usually treated with regimens that are a combination of the mentioned drugs along with dexamethasone (Yang et al. 2020; Cowan et al. 2022). For instance, CyBord is one of the regimens used for the treatment of individuals with MM which consists of bortezomib, cyclophosphamide, and dexamethasone (Yang et al. 2020; Cowan et al. 2022).

Mesenchymal stem cells (MSCs) are stem cells that have the potential for renewal and differentiation into desired types of cells and consequently tissues. Human MSCs have been broadly investigated in the field of regenerative medicine and for the modulation of the immune system, which highlights their broad range of applicability and potential. In 2021, Rodríguez-Fuentes and colleagues conducted an investigation and reported that 1,138 registered clinical trials were focusing on the clinical application of MSCs, with more than 60% of them being in Phase II (Rodríguez-Fuentes et al. 2021). Moreover, the most studied fields were reported to be immunology, traumatology, cardiology, and nephrology, and bone marrow (BM) was reported as the most applicable source for the isolation of MSCs (Rodríguez-Fuentes et al. 2021). Rodríguez-Fuentes et al. also reported that out of the clinical trials whose results had been officially published, the outcomes were satisfactory with no documented life-threatening adverse events (Rodríguez-Fuentes et al. 2021). Such clinical outcomes and ongoing investigations accentuate the importance of MSCs and MSC-derived therapeutics for the development of novel treatment modalities.

Microvesicles (MVs) are cell-secreted vesicles, which are 100–1000 nm in diameter, commonly produced and released by the cellular components of the tumor microenvironment (TME), especially malignant cells. Recently, accumulating evidence has demonstrated that TME-derived MVs play an important role in the emergence of drug resistance. For instance, Jaffar Ali and colleagues demonstrated that resistance to sorafenib in liver tumor cells is mediated by MVs through p53 attenuation and FOXM1 upregulation (Jaffar Ali et al. 2021). Other studies have also indicated that MVs are involved in tumor emergence and elevation of tumor malignancy (Bian et al. 2019). TME-derived MVs are highly stable; therefore, they can be isolated from patients and exploited for diagnostic purposes (Pontecorvi et al. 2020). It has been known that MVs extracted from MSCs could also mirror the therapeutic effects of their source cells to some extent, highlighting their potential in different fields of medicine. In 2020, Chen and colleagues demonstrated that MVs derived from MSCs could be used for the delivery of miR-100 and it was reported that these MVs could be used for the amelioration of acute lung injury in rat animal models (Chen et al. 2020). In 2019, Sabri and colleagues reported that bone marrow MSC-derived MVs show therapeutic benefits in the treatment of liver fibrosis in preclinical animal models induced using carbon tetrachloride (Sabry et al. 2019).

Aside from the discussed investigations, various other researchers have investigated the effects of bone marrow mesenchymal stem cell (BMSC)-derived MVs (hereafter referred to as BMSC-MVs) on the cell lines of MM. For instance, Dabbah and colleagues demonstrated that treatment of MM cell lines (namely, U266, ARP1, MM1S, and OPM2) with BMSC-MVs derived from normal donors significantly decreased cell viability, proliferation, migration, and translation initiation (Dabbah et al. 2017). According to the findings of an investigation by Roccaro and colleagues, it was found that normal BM mesenchymal stromal cell-derived exosomes suppressed the growth of MM cell lines whereas MM BM mesenchymal stromal cell-derived counterparts mediated a pro-proliferative effect (Roccaro et al. 2013). According to a report by Umezu and colleagues, it was demonstrated that exosomes derived from young BM stromal cells significantly suppressed the angiogenesis of MM cells in vitro (Umezu et al. 2017). Further inspection by Umezu and colleagues demonstrated a different expression profile of exosomal miRNA between exosomes derived from young and older BM stromal cells as the former exerted its antiangiogenic effects through the expression of miR-340 (Umezu et al. 2017). Aside from MM, researchers have also investigated the effects of BMSC-MVs on acute myeloid leukemia cell lines. For instance, Abbaszade Dibavar and colleagues demonstrated that treatment of the NB4 cell line with a combination of BMSC-MVs and arsenic trioxide significantly decreased cell viability, increased apoptosis rate, and decreased *Ki67* and *BCL-2* gene expression alongside and elevated *BAX* gene expression (Abbaszade Dibavar et al. 2018). Such findings underline the importance and potential of BMSC-MVs for different therapeutic purposes. In this study, MVs were isolated from human bone marrow BMSCs and characterized accordingly. Next, the effect of these BMSC-MVs on the MM cell line U266 was investigated.

## Materials and methods

### Cell culture

Human BMSCs were a kind gift from *Dr. Mahnoosh Abbaszade Dibavar* and her colleagues from their previous research projects in which these researchers had obtained the BMSCs from the *Stem Cell Technology Research Center* (Tehran, Iran) and characterized them as previously detailed (of note, these BMSCs were derived from the bone marrow aspirates of three healthy volunteers) (Abbaszade Dibavar et al. 2018); therefore, bone marrow aspirates were not collected and BMSCs were not isolated independently in this study. The BMSCs were cultivated in Dulbecco’s Modified Eagle’s Medium F-12 (DMEM/F-12; Gibco, United States) supplemented with 10% (v/v) fetal bovine serum (FBS; Gibco, United States) and penicillin/streptomycin (Gibco, United States). U266 cells were purchased from the Iranian Biological Resource Center (IRBC; Tehran, Iran) and cultured in RPMI 1640 culture medium (Gibco, United States) supplemented with 15% (v/v) FBS, penicillin/streptomycin, and L-glutamine. All cells were incubated in an incubator at 37 ºC in a humidified atmosphere with 5% CO_2_. The cultured cells were regularly counted and their viability was assessed using the Trypan blue staining method (Sigma-Aldrich, United States).

### BMSC-MV isolation

Human BMSCs were cultivated and subsequently trypsinized when reaching a confluency rate of ~ 80–85% and after three rounds of passing, cell culture supernatant was collected every 24 h for the next three rounds of passing. The collected supernatant was used for the isolation of the BMSC-MVs via ultracentrifugation at 20,000 × g for 1 h at 4 ºC after being centrifuged at 7,000 × g for 20 min for the removal of cell debris. Next, the supernatant was discarded and the BMSC-MVs were resuspended in phosphate-buffered saline (PBS) and stored at -80 ºC for further experiments.

### BMSC-MV characterization

The isolated microvesicles were assessed in terms of their morphology and size using transmission electron microscopy (TEM). Briefly, 50 µL of the BMSC-MVs diluted in PBS were placed on a formvar-coated grid, and after drying the samples, they were stained with uranyl acetate (2%). The microscopic images were eventually taken using a TEM instrument (CM20, Philips, United States). Moreover, to assess the concentration of the isolated microvesicles, the protein content of the BMSC-MVs was measured using a commercial kit (BCA protein quantification kit; cat. No. DB9684-50 ml; DNAbiotech, Tehran, Iran) as per the manufacturer’s instructions. To further characterize the BMSC-MVs, the dynamic light scattering (DLS) technique was employed to measure the diameter of the isolated microvesicles. In brief, the isolated microvesicles were diluted in 1 mL PBS, and the absorption of this suspension was measured at 630 nm via a Zetasizer (Zetasizer Nano ZS, Malvern, United Kingdom).

To further characterize the BMSC-MVs, a Western blotting experiment was conducted to verify the presence of specific markers CD63 (a characteristic marker of extracellular vesicles), CD73 (a mesenchymal stem cell marker), and CD81 (a characteristic marker of extracellular vesicles) (Sánchez-Abarca et al. 2016). To this aim, the BMSC-MVs were resuspended in lysis buffer (composition: 0.1% w/v sodium dodecyl sulfate, 1% Triton X-100, 50 mM Tris-HCl, 150 mM NaCl) and a protease inhibitor cocktail solution (Sigma-Aldrich, United States) as they were incubated on ice for 30 min. Following centrifugation at 12,000 × g for 15 min at 4 ºC for the removal of the non-soluble residues, the concentration of the protein samples was determined using the mentioned BCA protein quantification kit as per the manufacturer’s instructions. Next, the samples were subjected to sodium dodecyl-sulfate polyacrylamide gel electrophoresis (SDS-PAGE), and subsequently, the protein bands were transferred onto nitrocellulose membranes (UltraCruz® Nitrocellulose Pure Transfer Membrane; Santa Cruz Biotechnology; CA, United States) followed by blocking of the membranes using 5% non-fat dry milk in PBS with Tween 20 (0.5%; Sigma-Aldrich, United States) to prevent any non-specific binding. Next, the membranes were separately incubated with the following primary antibodies: rabbit polyclonal anti-human CD63 antibodies (cat. No. 11,271-T16; Sino Biological, China), rabbit monoclonal anti-human CD73 antibodies (cat No. ab124725; Abcam, MA, United States), and rabbit polyclonal anti-human CD81 antibodies (cat No. ab155760; Abcam, MA, United States) as per the suppliers’ instructions. Next, the membranes were incubated overnight at 4 °C. Following three rounds of washing with PBS, the membranes were incubated with horseradish peroxidase (HRP)-labeled mouse anti-rabbit antibodies (with a dilution ratio of 1:5000; cat. No. sc-2357; Santa Cruz Biotechnology, CA, United States) at room temperature for 1 h as per the supplier’s protocols. The membranes were visualized using enhanced chemiluminescence (ECL; cat. No. B111420; Pars Tous, Mashhad, Iran) and imaged subsequently. Of note, the BMSCs were also taken into consideration in the Western blotting experiments. Briefly, β-actin was used as the positive control as β-actin-reactive rabbit polyclonal antibodies (cat. No. ab8227; Abcam, MA, United States) were used as the primary antibodies and HRP-labeled mouse anti-rabbit antibodies as the secondary antibodies as per the suppliers’ instructions.

### Assessment of the BMSC-MV internalization into the U266 cells

To monitor the internalization of the BMSC-MVs into the target cells, the microvesicles were stained with carboxyfluorescein succinimidyl ester (CFSE; BioLegend, United States) as per the manufacturer’s instructions, and the treated cells were examined for the presence of the labeled microvesicles under a fluorescence microscope (Nikon Eclipse, Nikon, Japan). In brief, the isolated microvesicles were resuspended in 1 mL PBS and then 1 µL of CFSE was added to them as they were incubated at 37 ºC for 20 min in the dark. Next, 5 mL RMPI 1640 supplemented with 15% FBS was added to them as they were incubated at room temperature for 5 min in the dark. To remove any excess stain, the suspension was centrifuged at 20,000 × g for 1 h at 4 ºC. In the following, the supernatant was discarded and the microvesicles were resuspended in 3 mL RMPI 1640 supplemented with 15% FBS. Next, the CFSE-labeled BMSC-MVs were added to a 3 cm tissue culture plate in which U266 cells had already been seeded and then the cells were incubated. Ultimately, the internalization of the CFSE-labeled BMSC-MVs into the U266 cells was assessed under a fluorescence microscope.

### Assessing the viability of the U266 cells following their treatment with the BMSC-MVs via the MTT assay

To evaluate the viability of the U266 cells following their treatment with different concentrations of the BMSC-MVs, a 3-[4,5-dimethylthiazol-2-yl]-2,5 diphenyl tetrazolium bromide (MTT) assay was conducted. Briefly, 5 × 10^3^ U266 cells (resuspended in 50 µL RPMI 1640 and 50 µL PBS) were seeded into the wells of a flatbottom 96-well cell culture plate (SPL Life Sciences, South Korea). The cells were treated with either 0 (regarded as control), 15, 30, 60, or 120 µg/mL of the BMSC-MVs. Of note, blank wells were also taken into consideration for this experiment as they contained only RPMI and PBS (with a 1:1 ratio). The plates were then incubated in an incubator at 37 ºC with 5% CO_2_, and the viability of the cells was assessed via the MTT assay on Day 3 and Day 7. For the MTT assay, 10 µL of the MTT solution (10 mM) was added to the cells of each well, and the plate was incubated at 37 ºC with 5 CO_2_ for 4 h. After the incubation period, the plate was centrifuged at 100 × g at 24 ºC for 5 min, and then 70 µL of the supernatant was discarded. Next, 170 µL dimethyl sulfoxide (DMSO; 100 mM) was added to each well, and then the absorbance of each well was measured using a microplate reader instrument (Microplate Absorbance Reader 2020, Bichrom Anthos, France) at 570 nm.

### Assessing the cytotoxic effect of the BMSC-MV treatment on the U266 cells via the lactate dehydrogenase (LDH) assay

To assess the cytotoxic effect of the BMSC-MV treatment on the U266 cells, an LDH assay was conducted. To this aim, a commercial LDH cytotoxicity kit (CyQUANT™ LDH Cytotoxicity Assay; cat. No. C20300; Thermo Fisher Scientific, United States) was used as per the manufacturer’s instructions. The U266 cells were treated with 0, 15, 30, 60, or 120 µg/mL of the BMSC-MVs, and the cytotoxicity rate was measured on Day 3 and Day 7. Briefly, after the cultivation of the cells and their corresponding treatments, the cell culture supernatant of each well was transferred into the wells of a new 96‑well plate followed by the addition of the reaction mixture. After a 30-minute incubation at room temperature in the dark, the reactions were terminated by the addition of the Stop Solution, and the absorbance of the wells was measured at 490 nm. Of note, DMSO was used as the positive control and all of the obtained values were standardized accordingly.

### Assessment of the level of DNA damage following the treatment of the U266 cells with the BMSC-MVs

As an attempt to further investigate the cytotoxic effect of the BMSC-MVs on the U266 cell line, we proceeded to assess the level of cellular DNA damage following cell treatment using a commercial kit (Highly Sensitive 8‑OHdG Check ELISA; JaICA, Japan). This commercial kit utilizes a highly specific monoclonal antibody for the detection of 8‑hydroxy-2’-deoxyguanosine (8‑OHdG), a product of DNA damage. Briefly, the U266 cells were treated with 0, 15, 30, 60, and 120 µg/mL BMSC-MVs for 3 and 7 days, and after treatment, the supernatant of each cell culture was carefully collected by centrifugation at 1500 × g for 10 min at 4 ºC and used for the assessment of the level of 8‑OHdG as per the manufacturer’s instructions.

### Assessing the apoptosis of the U266 cells following their treatment with the BMSC-MVs

A flow cytometry assay using the annexin V/propidium iodide (PI) technique (FITC Annexin V Apoptosis Detection Kit; BD Biosciences, United States) was used for the evaluation of the apoptosis rate of the U266 cells following their treatment with 60 µg/mL of the BMSC-MVs for seven days. For this goal, 5 × 10^4^ U266 cells were seeded into the wells of a 6-well tissue culture plate (resuspended in 500 µL culture media and 500 µL PBS), treated with 60 µg/mL BMSC-MVs, and then incubated at 37 ºC with 5% CO_2_ for seven days. After the incubation, the cells were harvested and centrifuged at 300 × g for 5 min, after which the supernatant was discarded and the cells were washed twice with PBS, and ultimately resuspended in 100 µL binding buffer in separate tubes. Next, each tube was supplemented with 5 µL FITC annexin V and 10 µL PI as the tubes were incubated at room temperature in the dark for 15 min. Ultimately, 400 µL binding buffer was added to each tube and then the cells were analyzed using a flow cytometer instrument (BD FACSCalibur™, BD Biosciences, United States).

### Assessing the expression levels of *BCL2*, *BAX*, and *CCND1* in the U266 cells following their treatment with the BMSC-MVs

To further investigate the effect of the BMSC-MV treatment on the U266 cell line, the expression level of the anti-apoptotic gene *BCL2*, the pro-apoptotic gene *BAX*, and *CCND1* was evaluated using the qualitative real-time reverse-transcription PCR (qRT-PCR) technique. Of note, *GAPDH* was taken into consideration as the housekeeping gene in this experiment. For this goal, U266 cells were seeded into 6-well cell culture plates, treated with 60 µg/mL of the BMSC-MVs (resuspended in PBS), and then they were incubated at 37 ºC with 5% CO_2_ for seven days. After the incubation period, the cells were harvested and the total RNA was extracted from them. Briefly, the cells were centrifuged at 300 × g for 5 min, and then the supernatant was discarded as the cells were supplied with 1 mL Trizol reagent (TRIzol™ Reagent, Thermo Fisher Scientific, United States) and then incubated at room temperature for 5 min. Next, 200 µL ice-cold chloroform was added to each tube as the tubes were incubated at 4 ºC for 5 min. In the following step, the cells were centrifuged at 12,000 × g for 5 min at 4 ºC, and after observing the aqueous phase (containing RNA), the interphase, and the organic phase (containing DNA), the phase containing RNA was carefully extracted and moved to another tube. Next, each tube was supplied with isopropanol (the same volume as the aqueous phase solution), and the tubes were incubated at -80 ºC for 12–18 min. Further on, the tubes were centrifuged at 12,000 × g for 20 min at 4 ºC after being incubated at 4 ºC for 15 min. After centrifugation, the supernatant was discarded and the precipitated RNA was resuspended in ethanol (70%) as the tubes were centrifuged again at 12,000 × g for 15 min at 4 ºC. Next, the supernatant was discarded and the ethanol was evaporated after which the pelleted RNA was solubilized in diethylpyrocarbonate (DEPC)-treated water (Thermo Fisher Scientific, United States). Ultimately, the extracted RNA was quantified using a nanodrop instrument (NanoDrop 2000c, Thermo Scientific, United States) and also assessed by gel electrophoresis.

cDNA synthesis was performed using the AddScript cDNA Synthesis Kit (ADDBIO Inc., South Korea) as per the manufacturer’s instructions. The qRT-PCR assay was performed using a StepOne™ real-time PCR instrument (Applied Biosystems, Thermo Fisher Scientific, United States) and the primers used have been included in Table 1. Of note, 2^−∆∆Ct^ was applied for the calculation of the expression fold change of the investigated genes.

**Table 1:** Primers used for the assessment of *GAPDH*, *BAX*, *BCL2*, and *CCDN1* genes at the mRNA level

Primer	Sequence 5ˈ→ 3ˈ		Tm (ºC)	Product Size (bp)
GAPDH	Forward	CATGTTCGTCATGGGTGTGAAC	60	198
	Reverse	CACAGTCTTCTGGGTGGCAG		
BAX	Forward	CAAACTGGTGCTCAAGGC	60	204
	Reverse	CACAAAGATGGTCACCGTC		
BCL2	Forward	GTACTTAAAAAATACAACACAG	60	256
	Reverse	CTTGATTCTGGTGTTTCCC		
CCDN1	Forward	CAGAGTGATCAAGTGTGACCC	60	287
	Reverse	CGTCGGTGGGTGCAAGC		

### Statistical analysis

All experiments were carried out in triplicate and the data are presented as mean ± SD. One-way ANOVA with Student’s T test or Tukey’s multiple comparisons tests was used for statistical comparison between the experimental groups, and a p-value < 0.05 was considered statistically significant. Statistical analyses and plot illustrations were carried out using the GraphPad Prism software (version 9.0.5; GraphPad Software, United States).

## Results

### BMSC culture and BMSC-MV characterization

**Fig. 1:**
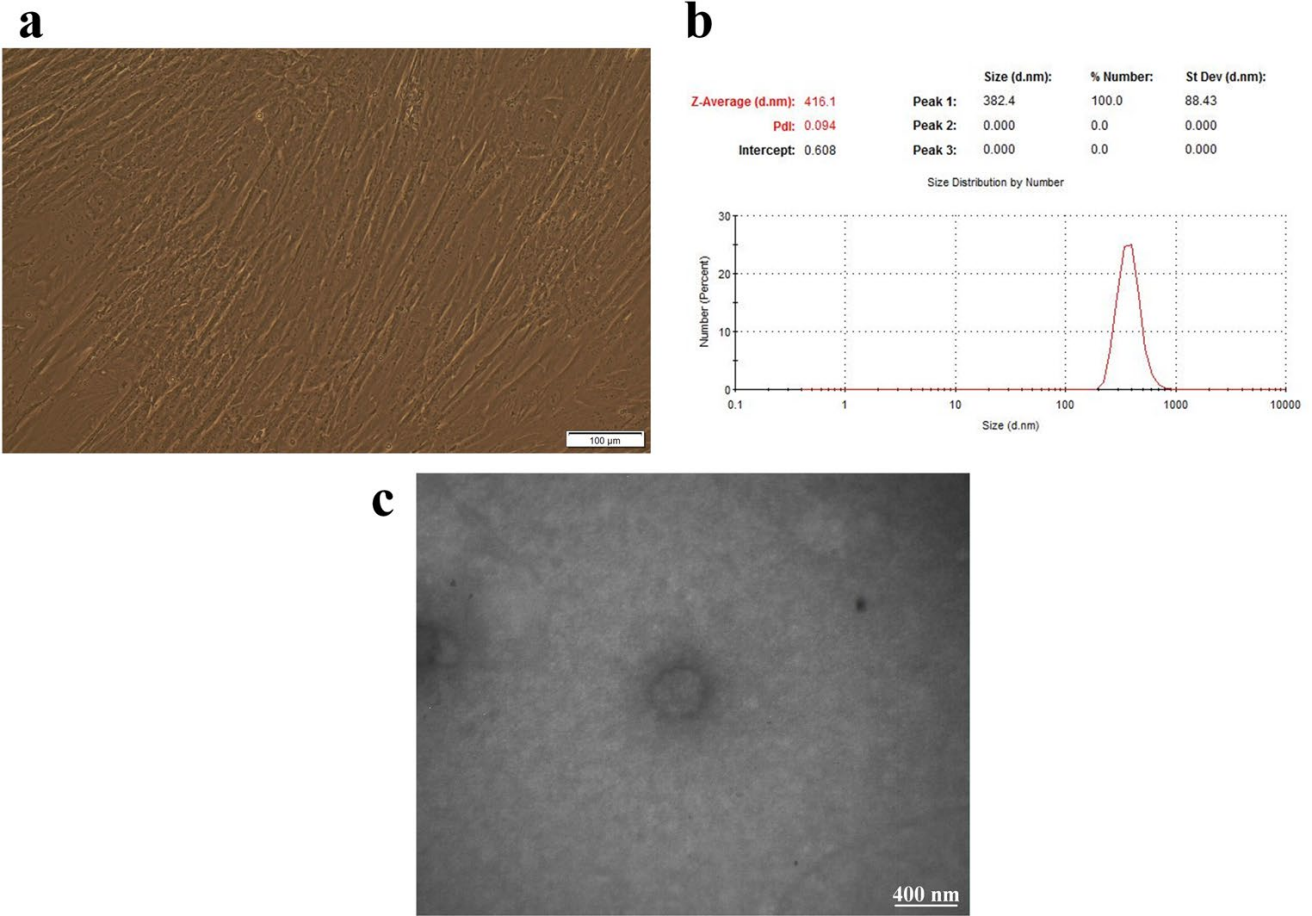
The morphology of the BMSCs and characterization of the BMSC-MVs. **a**: The BMSCs micrographed using a phase contrast microscope. **b**: Dynamic light scattering (DLS) results of the BMSC-MVs indicating an average size of around 416 nm. **c**: A TEM image of the BMSC-MVs

The isolated BMSCs were verified through their spindle-shaped morphology using phase contrast microscopy (Fig. 1a) and the expression of known BMSC surface receptors, namely CD73, CD90, CD105, and CD44, as previously reported by Abbaszade Dibavar and colleague (Abbaszade Dibavar et al. 2018). These BMSCs were cultured and after three rounds of passing, their supernatant was used for the collection of the BMSC-MVs. Regarding the characterization of the isolated BMSC-MVs, the results of the DLS technique indicated an average size of around 416 nm, confirming that the isolated particles are MVs (Fig. 1b). Moreover, the TEM technique was also employed for evaluating the shape and quality of the isolated BMSC-MVs. According to the results, the BMSC-MVs demonstrated an average size of around 400 nm with their membranes intact even after the process of MV isolation (Fig. 1c).

**Fig. 2:**
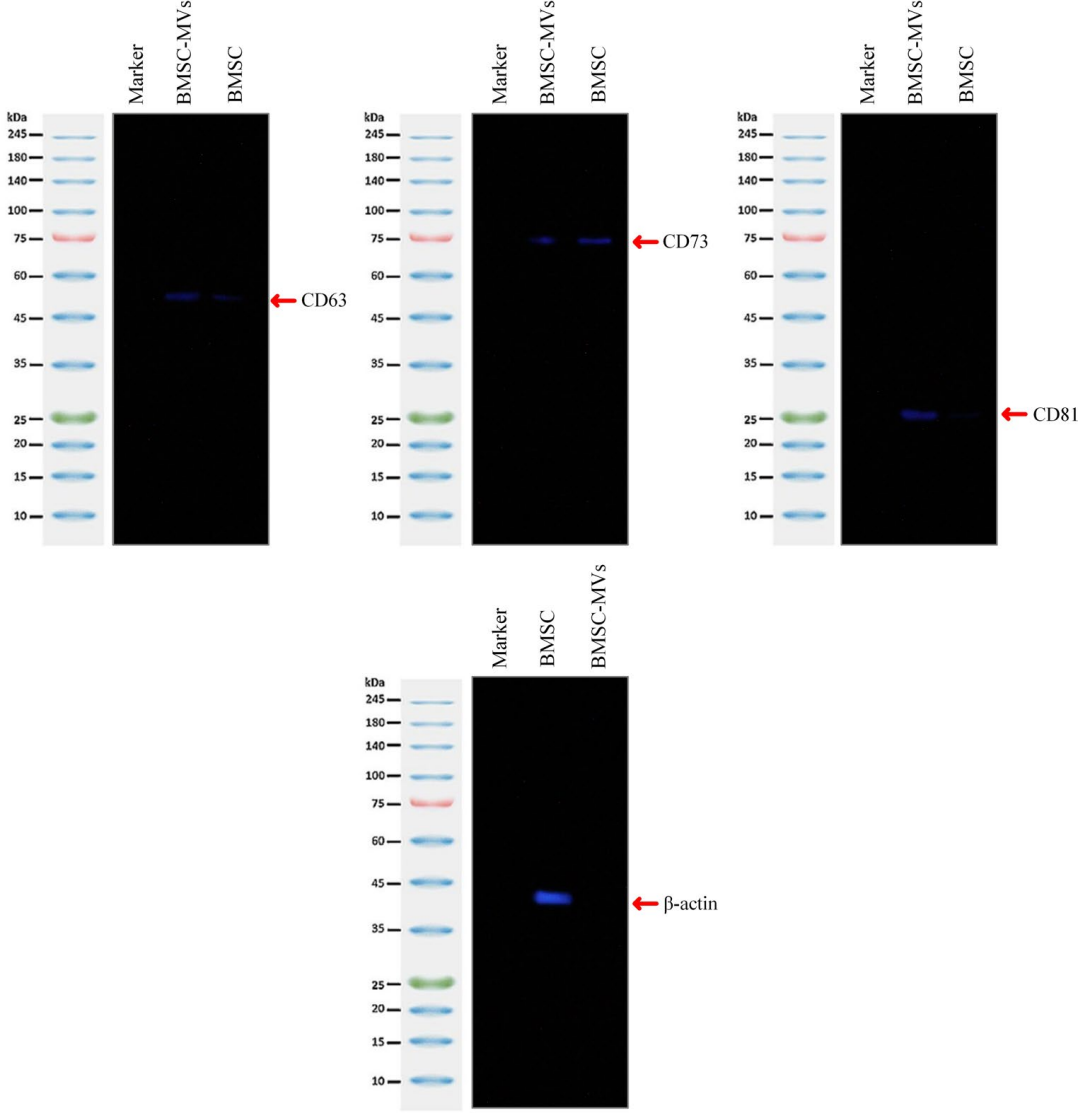
Western blotting assay for the verification of the BMSC-MVs. CD63, CD73, and CD81 with approximately 50, 75, and 26 kDa protein bands, respectively, were detected

The results of the Western blotting assay confirmed the presence of the MV-specific biomarkers CD63 and CD81, which were expressed at a relatively higher level in comparison with those of the BMSCs, their parental cells (Fig. 2). Moreover, inspection for the expression of CD73 demonstrated that the BMSC-MVs expressed this mesenchymal marker; however, at a relatively lower level as compared with the BMSCs (Fig. 2).

### Assessment of the BMSC-MV internalization into the U266 cells

**Fig. 3:**
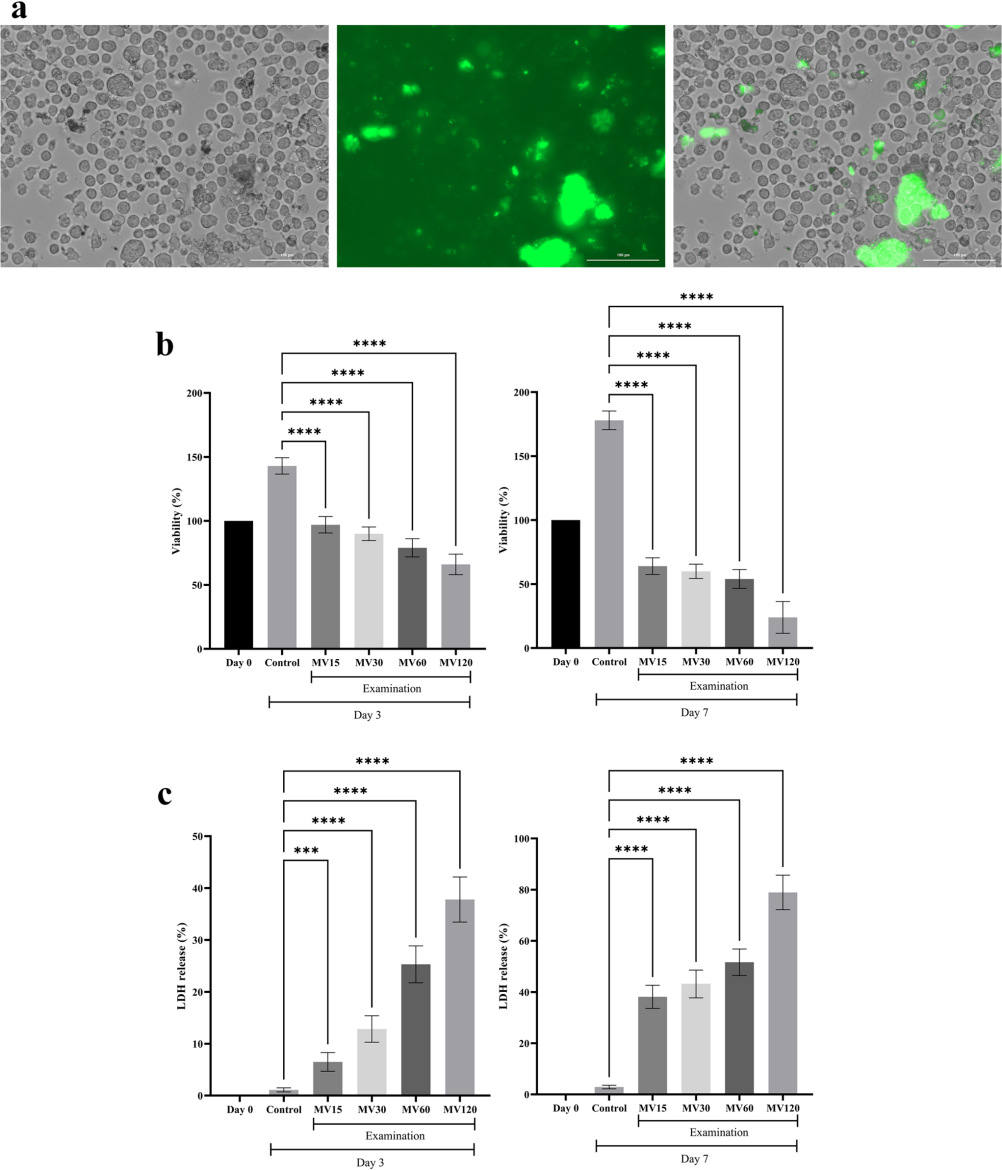
Internalization of the BMSC-MVs into the U266 cells and the effect of the BMSC-MV treatment on the U266 cells. **a**: Detection of the CSFE-labeled BMSC-MVs internalized into the U266 cells under a fluorescence microscope. Bright-field (left), fluorescence (middle), and merged images (right). **b**: Assessment of the viability of the U266 cells via the MTT assay following their treatment with 0, 15, 30, 60, and 120 µg/mL BMSC-MVs on Day 0, Day 3, and Day 7. **c**: Assessment of the cytotoxicity of the treatment of the U266 cells with 0, 15, 30, 60, and 120 µg/mL BMSC-MVs on Day 0, Day 3, and Day 7 as assessed via the LDH assay. *** and **** represent *p* < 0.001 and *p* < 0.0001, respectively. All experiments were carried out in triplicate (*n* = 9)

According to the results of the Trypan blue assay, the viability of the U266 cells was determined more than 96 ± 1% (data not shown). Moreover, using a fluorescence microscope, it was demonstrated that the BMSC-MVs were sufficiently internalized into the U266 cells following treatment as presented in Fig. 3a.

### Viability of the U266 cells following their treatment with the BMSC-MVs

According to the results of the MTT assay, the viability of the U266 cells decreased three and seven days following their treatment with the BMSC-MVs in a dose-dependent fashion (Fig. 3b). On Day 3, the viability of the U266 cells treated with 15 µg/mL BMSC-MVs did not demonstrate a significant decline in comparison with the viability of U266 cells on Day 0 (*p* > 0.05). However, treatment of the U266 cells with 30, 60, and 120 µg/mL BMSC-MVs resulted in significantly lower viability rates in comparison with that of Day 0 (*p* < 0.05, *p* < 0.0001, and *p* < 0.0001, respectively). In comparison with the viability of the U266 cells of the control group (which received no BMSC-MV treatment) on Day 3, the viability of the U266 cells treated with 15, 30, 60, and 120 µg/mL BMSC-MVs demonstrated a significant decline in a dose-dependent manner, as the lowest viability was observed in the 120 µg/mL BMSC-MVs treatment group (*p* < 0.0001 for all comparisons).

On Day 7, all of the treated groups demonstrated a significant decline in their viability rates in comparison with the viability of the U266 cells on Day 0 (*p* < 0.0001 for all comparisons). Moreover, as compared with the viability of the U266 cells of the control group on Day 7, the lowest viability rate was observed in the treated cells of the 120 µg/mL BMSC-MV group (*p* < 0.0001) followed by the viability of the U266 cells in the groups treated with 60 µg/mL BMSC-MVs, 30 µg/mL BMSC-MVs, and 15 µg/mL BMSC-MVs (*p* < 0.0001 for all groups). These findings demonstrate that increasing the duration of treatment and dose of the BMSC-MVs correlates with lower viability rates in the treated U266 cells.

### Cytotoxic effect of the BMSC-MV treatment on the U266 cells

The results of the LDH assay corroborated those of the MTT assay as treatment of the U266 cells with the BMSC-MVs resulted in significant cytotoxicity against the treated cells (Fig. 3c). According to the results, On Day 3, the highest rate of cytotoxicity was observed in the experimental group in which the U266 cells were treated with 120 µg/mL BMSC-MVs, followed by those treated with 60, 30, and 15 µg/mL BMSC-MVs which were significantly higher as compared with those of the control (with no treatment) (*p* < 0.0001 for all comparisons, except for the 15 µg/mL BMSC-MV treatment group vs. the control group which was *p* < 0.001). Moreover, a similar pattern of cytotoxicity was observed for all of the experimental groups treated with the BMSC-MVs on Day 7, as the highest rate of cytotoxicity occurred in the U266 cells treated with 120 µg/mL BMSC-MVs, followed by those treated with 60 and 30 µg/mL BMSC-MVs, and the lowest rate was observed in the cells treated with 15 µg/mL BMSC-MVs. All of these values were significantly higher in comparison with those of the control groups (*p* < 0.0001 for comparisons).

### Assessment of the level of 8‑OHdG following the treatment of the U266 cells with the BMSC-MVs

**Fig. 4:**
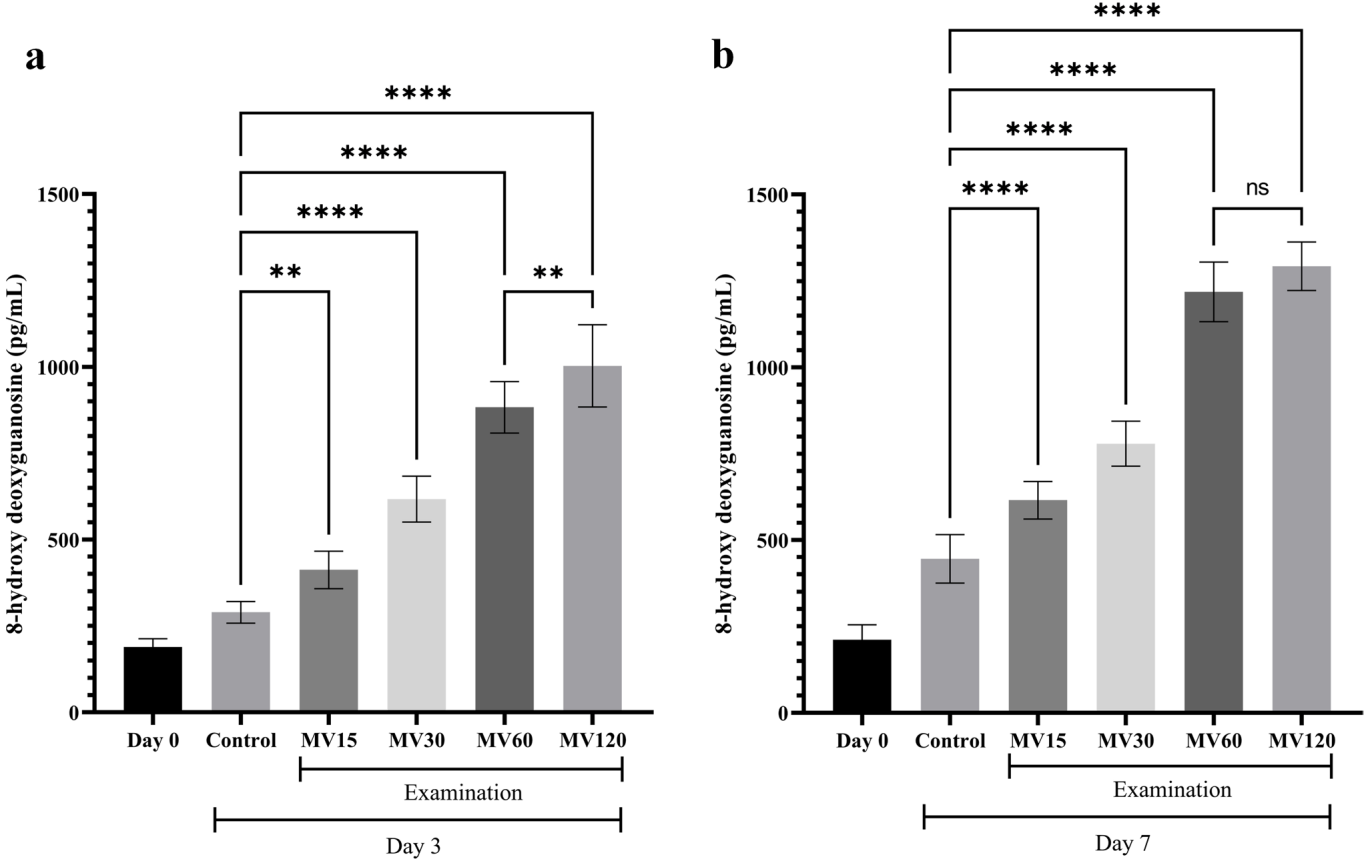
Assessment of the 8‑OHdG level in the U266 cells following their treatment with 0, 15, 30, 60, and 120 µg/mL BMSC-MVs on Day 0, Day 3 (**a**), and Day 7 (**b**). ** for *p* < 0.001, **** for *p* < 0.0001, and ns for *p* > 0.05. All experiments were carried out in triplicate (*n* = 9)

According to the obtained results, treatment of the U266 cells with the BMSC-MVs significantly increased the level of DNA damage, as reflected by the measured amount of 8‑OHdG, in the treated cells in comparison with those of the untreated control cells (Fig. 4). On Day 3, the highest level of DNA damage was observed in the U266 cells treated with 120 µg/mL BMSC-MVs, followed by the cells treated with 60, 30, and 15 µg/mL BMSC-MVs, all of which were significantly higher than those of the untreated control groups (*p* < 0.0001, *p* < 0.0001, *p* < 0.0001, and *p* < 0.01, respectively) (Fig. 4a). As the treatment period was extended from 3 days to 7 days, the level of DNA damage increased in the cells of the experimental groups (Fig. 4b). Briefly, it was demonstrated that all of the treated cell groups exhibited significantly higher levels of 8‑OHdG in comparison with those of the untreated control group as the U266 cells treated with 120 µg/mL BMSC-MVs exhibited the highest level of 8‑OHdG, followed by those treated with 60, 30, and 15 µg/mL BMSC-MVs (*p* < 0.0001 for all comparisons). A comparison between the level of 8‑OHdG in the 120 µg/mL BMSC-MV treatment group with that of the 60 µg/mL BMSC-MV treatment group demonstrated comparable levels of 8‑OHdG after 7 days of treatment (*p* > 0.05), unlike what was observed in the comparison of these two experimental groups after 3 days of treatment (*p* < 0.01).

### Apoptosis of the U266 cells following their treatment with the BMSC-MVs

**Fig. 5:**
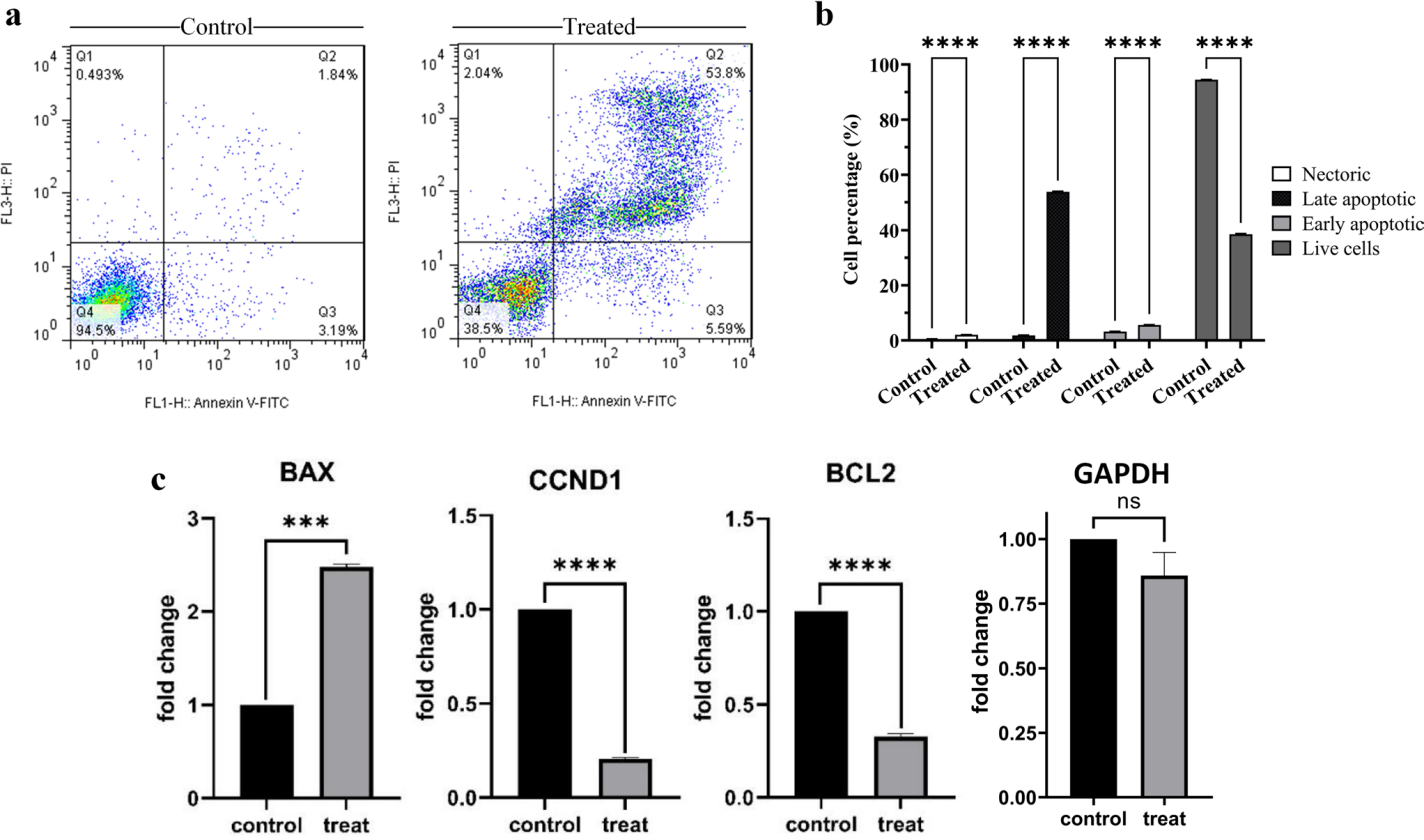
The effect of the BMSC-MV treatment on the apoptosis rate and the *BAX*, *BCL2*, *CCND1*, and *GAPDH* mRNA expression levels of the U266 cells. **a**: Annexin V/PI flow cytometric analysis for the assessment of the apoptosis rate of the U266 cells following their treatment with 60 µg/mL BMSC-MVs for seven days. Q1, Q2, Q3, and Q4 respectively represent the percentage of necrotic, late apoptotic, early apoptotic, and live cells in the scatter plots. **b**: Statistical comparison between the percentage of the necrotic, late apoptotic, early apoptotic, and live cell U266 cells following their treatment with 60 µg/mL BMSC-MVs for seven days in comparison with those of the control group. **c**: Assessment of the expression of the *BAX*, *BCL2*, *CCND1*, and *GAPDH* genes at the mRNA level in the U266 cells following their seven-day treatment with the BMSC-MVs. *** for *p* < 0.001, **** for *p* < 0.0001, and ns for *p* > 0.05. All experiments were carried out in triplicate (*n* = 3)

To investigate the effect of the BMSC-MV treatment on the apoptosis rate of the U266 cells, the target cells were treated with 60 µg/mL BMSC-MVs for seven days and then their apoptosis rate was analyzed using the annexin V/PI technique via a flow cytometer. In the flow cytometric analysis, Q1, Q2, Q3, and Q4 represent the percentage of necrotic, late apoptotic, early apoptotic, and live cells, respectively. According to the results (Fig. 5a and **b**), the percentage of necrotic, late apoptotic, and early apoptotic U266 cells significantly increased in the BMSC-MV-treated cell group in comparison with those of the control group (*p* < 0.0001 for all comparisons). Moreover, it was also demonstrated that the percentage of the live U266 cells significantly declined following their treatment with 60 µg/mL BMSC-MVs for seven days (~ 38% live cells) in comparison with that of the control group (~ 94% live cells) (*p* < 0.0001). These findings demonstrated that BMSC-MV treatment can significantly induce apoptosis in the U266 cells.

### *BCL2*, *BAX*, and *CCND1* expression levels following the BMSC-MV treatment

The effect of treatment with 60 µg/mL BMSC-MV on the expression level of the anti-apoptotic gene *BCL2*, pro-apoptotic gene *BAX*, and *CCND1* was investigated in the U266 cells at the mRNA level using the qRT-PCR technique. The findings indicated deviance in the expression rate of all of the studied genes in favor of apoptosis in the treated cell group (Fig. 5c). In brief, the expression of the *BAX* gene significantly increased in the BMSC-MV-treated U266 cells by more than two-fold (*p* < 0.001) whereas the expression of the *CCND1* and *BCL2* genes significantly decreased in the treated cells (*p* < 0.0001 for both groups) seven days following the BMSC-MV treatment. Regarding the expression level of the housekeeping *GAPDH* gene, even though the treatment of the U266 cells with the BMSC-MVs resulted in a reduction in the expression of the *GAPDH* gene at the mRNA level in comparison with that of the untreated cells, this difference was not statistically significant (*p* > 0.05).

## Discussion

Cell-based therapies and stem cell-based therapies have now been known as the hot topic for the development of novel treatment modalities against a wide range of malignancies. Owing to the characteristics of stem cells, such as self-renewal and differentiation into different types of cells, these cells have attracted the attention of researchers for the development of potential treatments against diseases resistant to conventional therapies. Stem cells are currently used in other fields of research including tissue regeneration. Among different types of stem cells, MSCs can be named the most investigated type of stem cells to date. MSCs are mature non-hematopoietic stem cells that play important roles in the proliferation, implantation, differentiation, and self-renewal of cells through the production and secretion of certain growth factors and MVs (Kulus et al. 2021). MSCs can be isolated from the tissues of mesodermal origin, and they contribute to the regeneration of several tissues, including bone and cartilage. Researchers have reported MSC isolation from numerous sources which include Wharton’s jelly, umbilical cord, bone marrow, placenta, and adipose tissue, from which bone marrow seems to be one of the most available sources. Typically, MSCs have the potential to differentiate into non-mesenchymal cells (namely, hepatocytes or neurons) or mesenchymal cells (namely, adipocytes, osteoblasts, and chondrocytes). Over the past decade, researchers have focused on investigating the therapeutic effect of MSCs against blood-based malignancies (Mishra et al. 2020; Han et al. 2019). One of the recent studies in this regard was conducted by Fathi et al. in which the researchers investigated the effects of BMSCs on a chronic myeloid leukemia cell line, K562 (Fathi et al. 2019). These researchers demonstrated that treatment with BMSCs culminated in significantly higher apoptosis rates and cell cycle arrest in the target cell lines in comparison with those of the control group (Fathi et al. 2019). Moreover, it was also reported that inhibition of the proliferation of K562 cells was mediated by the secretion of certain cytokines (namely, TIMP-1 and CINC-1) from BMSCs following their co-cultivation (Fathi et al. 2019). These researchers also reported that G0/G1 cell cycle arrest and elevated apoptosis rate of K562 cells were mediated by an increase in the expression of the apoptosis-related gene *BAX* and a decline in the expression of *BCL2* (Fathi et al. 2019). Based on the findings of Yang et al., MSCs derived from adipose tissues were capable of mediating growth inhibition in a range of cancer cell lines of the lung, glioma, breast, and colorectal origin (A549, U251, MCF-7, and HT29, respectively) (Yang et al. 2014). In 2014, Bozok Cetintas and colleagues reported the findings of an investigation focused on the effects of MSCs on the growth, cell death, and gene expression of an acute lymphoblastic leukemia (ALL) cell line (Bozok Cetintas et al. 2014). In brief, these researchers co-cultivated MSCs with the CCRF-CEM cell line for three days and observed a significant incremental pattern in the expression of *BAX*, *p53*, *caspase 9*, and various growth factors (namely, *EGFR* and *VEGF*) alongside a decreased CCRF-CEM cell proliferation rate (Bozok Cetintas et al. 2014). Ultimately, the investigators attributed the elevated apoptosis rate of the CCRF-CEM cells to *p53/BAX* (Bozok Cetintas et al. 2014). According to a 2010 report by Secchiero and colleagues, it was demonstrated that human BMSCs exert tumoricidal effects against two distinct disseminated non-Hodgkin’s lymphoma xenografts (aggressive EBV + B lymphoblastoid and indolent EBV + Burkitt lymphoma) in SCID mice (Secchiero et al. 2010). Researchers have also investigated rat umbilical cord stem cells as potential therapeutics against mammary carcinomas and reported that these cells are capable of mediating complete tumor outgrowth suppression and tumor disappearance a hundred days following tumor establishment without clinical signs of tumor recurrence or metastasis (Ganta et al. 2009). Based on the finding of a study by Cousin and co-investigators, human adipose tissue-derived adult stromal cells suppressed the proliferation of pancreatic tumor cells in vitro (on prostate cancer, liver cancer, and colorectal cancer cell lines) and mediated prolonged and pronounced tumor regression in pancreatic adenocarcinoma mouse models following localized administration (Cousin et al. 2009).

Researchers have also applied MSCs to other fields, other than for the treatment of oncological indications. For instance, according to a recent report by Hashem Boroojerdi and colleagues, it was demonstrated that MSCs could be applied to increase the population of hematopoietic stem cells in vitro enabling higher quantities for clinical application and obviating the need for hazardous manipulation of hematopoietic stem cells (Hashem Boroojerdi et al. 2022). In 2018, Ferreira and co-investigators experimented on the effects of BMSCs on the resolution of nerve damage in individuals with diabetes and reported that MSCs contribute to the blockade of the neuroinflammatory cascades of the spinal cord (Evangelista et al. 2018). According to a report by Cui and co-investigators, human umbilical cord MSCs amend cognitive function in preclinical mouse models of Alzheimer’s disease through hippocampal neurogenesis and oxidative stress reduction (Cui et al. 2017). Despite offering therapeutic benefits against a wide range of oncological malignancies and disorders, MSCs face multiple hindrances that limit their broader application in the clinical aspect. For instance, safety issues relating to the administration of MSCs and the possible risks of graft-versus-host disease (GvHD) could be of paramount importance (Si et al. 2011). Moreover, since MSCs are living therapeutics, there might be concerns regarding their proliferation and biological stability in the prospective recipients (Si et al. 2011). Researchers have found an alternative for leveraging the therapeutic benefit of MSCs in clinics by using MSC-derived MVs, such as those isolated and characterized in the current study. MVs, alternatively known as microparticles or ectosome, are produced and secreted by a vast majority of cells physiologically or even under critical or abnormal conditions, which might include stress, apoptosis, or emergence of malignancy. Such MVs can fuse with the membrane of their corresponding target cells, thereby delivering their constituent into the target cells. Researchers have attributed various functions and roles to MVs which are expectedly dependent on their content, which themselves are dependent on the type of cell from which the MVs have been secreted. For instance, since MSCs are capable of mediating anti-inflammatory responses, immune system modulation, neurogenesis, and tissue damage repair, MVs derived from MSCs share similar capabilities as it has been demonstrated that MVs derived from tumor cells are capable of inducing tumor aggressiveness due to the containment of matrix metalloproteases. MVs can establish a connection between cells due to their content which is an important occurrence, especially when MVs are derived from tumor cells and affect healthy cells. MVs offer some advantages over cell-based therapeutics including their low rate of toxicity and immunogenicity, their ability to cross the blood-brain barrier (BBB), and containment of endogenous bioactive compounds protected from degradation (Mohd Noor et al. 2021; Favaro et al. 2014). As previously discussed, researchers have put a tremendous amount of time and effort into investigating the effect of MVs derived from MSCs on the characteristics, proliferation rate, and survival of various malignant cells. Herein, we focused on assessing the potential of human BMSC-MVs in reducing the survival rate of the MM cell line U266. The hypothesis was formed based on the fact that no definitive treatment modality has been found for MM and the fact that the treatment of MM patients with chemotherapy and other classes of therapeutics has been entwined with adverse effects on the vital organs of the patients. In this study, the effect of BMSC-MVs on the U266 cells was investigated for seven days, and the findings indicated a significant reduction in the expression of the *BCL2* and *CCND1* genes in comparison with those of the control group, whereas a significant increase in the expression of the *BAX* gene was observed at the mRNA level. These findings indicated that treatment of the U266 cells with BMSC-MVs results in increased apoptosis and decreased proliferation in the target cells.

Apoptosis is a physiological process in which cells undergo a series of successive events resulting in programmed cell death without the release of damaging cellular substances, such as proteases, to the neighboring environment (Elmore 2007). Since aged cells and malignant cells are principally eliminated through apoptosis, this process is considered an important key in the maintenance, regulation, and homeostasis of the body, and failure in the induction of apoptosis culminates in the emergence of immortal malignant cells (Elmore 2007). Apoptosis occurs due to distinct reasons including receptor-ligand bindings, lack of particular growth factors, or detrimental gene damage (Elmore 2007). Mainly, apoptosis is initiated upon the activation of a particular group of enzymes, known as caspases. Caspases are initially produced as inactive precursor proteins that undergo proteolytic cleavage once a death signal is received by the cell, and caspase-3 is an important key player in the induction of apoptosis since it carries out certain proteolytic cleavages of important downstream cellular proteins (Fan et al. 2017). Briefly, the intracellular amount of pro-apoptotic molecules, such as BAX, or anti-apoptotic molecules, such as BCL2, corresponds to the level of cell sensitivity to apoptosis (Fan et al. 2017). The BCL2 family has distinct members, some of which act in favor of apoptosis induction and some are potent inhibitors of apoptosis (Fan et al. 2017). While BAX, a BCL2 antagonist, is located within the cytoplasm or cell membrane, BCL2 is located within the mitochondria and cell nucleus (Fan et al. 2017). What is commonly known as a *death signal* activates BAX which leads to the subsequent release of cytochrome C from the cell mitochondria and binding of apoptotic protease activating factor (Apaf-1) to Caspase-9 (Fan et al. 2017). These occurrences culminate in cell death through the activation of the caspase cascade (Fan et al. 2017). In the current study, the expression level of the *BCL2* gene by the U266 cells significantly declined, compared to the control group, following their treatment with the BMSC-MVs for seven days whereas *BAX* expression significantly increased. This means that the BMSC-MV treatment has a significant impact on the induction of apoptosis in the treated cells and this proposes their application for antitumor purposes.

Numerous studies have investigated the role of *BCL2* and *BAX* in cell survival. According to a study by Hong et al., it was elucidated that bee venom-induced apoptosis in human rheumatoid synovial fibroblasts is mediated by elevated expression of *BAX* and *caspase-3* and decreased expression of *BCL2* (Hong et al. 2005). According to another study, Perlman and co-investigators demonstrated that the apoptosis of prostate epithelial cells is correlated with the expression level of *BAX* and *BCL2* as elevation in the expression of the former coincides with the onset of apoptosis (Perlman et al. 1999). Such findings accentuate the importance of the *BAX*: *BCL2* expression ratio in the apoptosis process. Our results are also consistent with these findings as it was demonstrated that elevated expression of *BAX* and a decrease in the expression of *BCL2* induced apoptosis in the U266 cells following their seven-day treatment with 60 µg/mL BMSC-MVs.

Cyclin D1 is a 45 kDa protein encoded by the human *CCND1* gene and it plays an important role in the regulation of cell cycle transition from the G1 to S phase (Tchakarska and Sola 2020). Cyclin D1 forms complexes with CDK4 and CDK6 proteins and mediates the phosphorylation of the retinoblastoma protein, upon which the E2F transcription factor is activated and the cell cycle enters the S phase (Tchakarska and Sola 2020). The overexpression of cyclin D1 is associated with a shortened G1 cell cycle phase and decreased dependency of cells on growth factors (Tchakarska and Sola 2020). Under such circumstances, normal cell cycle regulation is lost leading to unhinged cell proliferation, which is deemed a key step in the development and emergence of malignant cells (Tchakarska and Sola 2020; Barnes and Gillett 1998). Elevated expression of *CCND1* has also been hypothesized by Hoogstraat and colleagues to induce therapy resistance based on the findings of an investigation in which breast tumor samples were characterized before and after treatment to investigate the potential underlying mechanisms of chemotherapy resistance (Hoogstraat et al. 2022). Accumulating evidence has also suggested that elevated expression of the *CCDN1* gene has been documented in numerous oncological indications which brands it a “*negative prognostic marker*” (Montalto and Amicis 2020). Based on the findings of a 2017 report by Ahlin and colleagues, it was demonstrated that only in patients with ER + breast cancer (and not in those with ER- disease), the elevated expression of cyclin D1 is correlated with increased cell proliferation and a three-fold higher death risk (Ahlin et al. 2017). It has also been reported that elevated expression of the *CCND1* gene is associated with an increment in the resistance of breast cancer tumors to hormone therapies (Alao 2007). Our findings indicated a significant decrease in the expression rate of *CCND1* at the mRNA level in the U266 cells following their treatment with the BMSC-MVs for seven days in comparison with that of the control group, which implies that the BMSC-MV treatment could be regarded as therapeutically beneficial, consistent with other researchers’ findings.

Since the microenvironment of leukemic bone marrow is populated with both leukemic cells and normal cells, it is hypothesized that BMSC-MVs might influence leukemic cells of the bone marrow microenvironment by positively or negatively affecting disease progression. Researchers have recently dedicated a great deal of effort and time to investigating the direct and indirect effects (through the secretion of soluble factors) of MSCs on leukemic cells, which have yielded interesting results. According to Ramasamy et al., the proliferation of malignant hematopoietic and non-hematopoietic cells is similarly suppressed by MSCs, and MSCs induce transient G1 phase cell cycle arrest (Ramasamy et al. 2007). However, Ramasamy et al. reported contradictory in vivo results as the administration of tumor cells and MSCs into preclinical mouse models resulted in a higher tumor cell proliferation rate in comparison with that of the animals only receiving tumor cells (Ramasamy et al. 2007). Based on such contradictory in vivo and in vitro findings, Ramasamy et al. stated that the clinical application of MSCs in patients with oncological indications must be carried out carefully (Ramasamy et al. 2007). Our findings could shed more light on the therapeutic effect of the BMSC-MVs on malignant cells; however, in-depth in vitro and in vivo studies must be conducted to conclude more confidently. The limitations of this study include investigating the durability of the effect of the BMSC-MVs on the U266 cells, investigating the effects of the BMSC-MV treatment on the U266 cells for a longer duration, and studying the effects of the BMSC-MV treatment on the U266-based MM mouse models to observe if similar results can be obtained.

## Conclusion

In a nutshell, it can be asserted that a seven-day treatment of the U266 cells with the BMSC-MVs increases their apoptosis through increasing *BAX* expression and decreasing *BCL2* and *CCND1* expression. Our findings could shed more light on the effect of the BMSC-MVs on the survival of the U266 cells, thus offering BMSC-MVs as potentially applicable therapeutics against MM; however, carefully conducted preclinical and clinical investigations are warranted beforehand.

## Data Availability

All data generated or analysed during this study are included in this published article [and its supplementary information files].

## Abbreviations

MM: Multiple myeloma
CAR: Chimeric antigen receptor
MSCs: Mesenchymal stem cells
BM: Bone marrow
MVs: Microvesicles
TME: Tumor microenvironment
BMSC: Bone marrow mesenchymal stem cell
BMSC-MVs: bone marrow mesenchymal stem cell-derived MVs
DMEM: Dulbecco’s Modified Eagle’s Medium
FBS: Fetal bovine serum
IRBC: Iranian Biological Resource Center
PBS: Phosphate-buffered saline
TEM: Transmission electron microscopy
DLS: Dynamic light scattering
SDS-PAGE: Sodium dodecyl-sulfate polyacrylamide gel electrophoresis
HRP: Horseradish peroxidase
ECL: Enhanced chemiluminescence
CFSE: Carboxyfluorescein succinimidyl ester
MTT: 3-[4,5-dimethylthiazol-2-yl]-2,5 diphenyl tetrazolium bromide
DMSO: Dimethyl sulfoxide
LDH: Lactate dehydrogenase8OHdG, 8hydroxy-2’-deoxyguanosine
PI: Propidium iodide
qRT-PCR: Qualitative real-time reverse-transcription PCR
DEPC: Diethylpyrocarbonate
ALL: Acute lymphoblastic leukemia
GvHD: Graft-versus-host disease
BBB: Blood-brain barrier
Apaf-1: Apoptotic protease activating factor
